# Diagnostic performance of peroxiredoxin 1 to determine time-of-onset of acute cerebral infarction

**DOI:** 10.1038/srep38300

**Published:** 2016-12-06

**Authors:** Sébastien Richard, Vanessa Lapierre, Nicolas Girerd, Mathieu Bonnerot, Pierre R. Burkhard, Linnéa Lagerstedt, Serge Bracard, Marc Debouverie, Natacha Turck, Jean-Charles Sanchez

**Affiliations:** 1 Department of Neurology, Stroke Unit, University Hospital of Nancy, 54035 Nancy, France; 2 Centre d′Investigation Clinique Plurithématique CIC 1433, University Hospital of Nancy, 54500 Vandoeuvre-lès-Nancy, France; 3 Department of Human Protein Sciences, University Medical Center, 1206 Geneva, Switzerland; 4 Department of Neurology, University Hospital of Geneva, 1205 Geneva, Switzerland; 5 Department of Neuroradiology, University Hospital of Nancy, 54035 Nancy, France

## Abstract

Accurately determining time-of-onset of cerebral infarction is important to clearly identify patients who could benefit from reperfusion therapies. We assessed the kinetics of peroxiredoxin 1 (PRDX1), a protein involved in oxidative stress during the acute phase of ischemia, and its ability to determine stroke onset in a population of patients with known onset of less than 24 hours and in a control group. Median PRDX1 levels were significantly higher in stroke patients compared to controls. PRDX1 levels were also higher from blood samples withdrawn before *vs.* after 3 hours following stroke onset, and before *vs*. after 6 hours. ROC analysis with area under the curve (AUC), sensitivity (Se) and specificity (Sp) determined from the Youden index was performed to assess the ability of PRDX1 levels to determine onset. Diagnostic performances of PRDX1 levels were defined by an AUC of 69%, Se of 53% and Sp of 86% for identifying cerebral infarction occurring <3 hours, and an AUC of 68%, Se of 49% and Sp of 88% for cerebral infarction occurring <6 hours. These first results suggest that PRDX1 levels could be the basis of a new method using biomarkers for determining cerebral infarction onset.

While cerebrovascular biomarkers are widely used to diagnose, define the severity and predict the prognosis of patients with cerebral ischemia^1^,^2^, only one study has assessed their kinetics at the acute phase of stroke^3^. However, this could be an interesting field of research as many proteins are synthetized, released and activated at the acute phase of stroke. Moreover, it would help to answer an important clinical question: how to accurately determine the time-of-onset of acute ischemic stroke in patients of unknown onset so as to perform reperfusion therapies within recommended time windows. In current clinical practice, physicians attempt to determine time of onset using cerebral magnetic resonance imaging (MRI) and the diffusion/fluid attenuated inversion recovery (FLAIR) mismatch. However, this method is not completely reliable^4^. Furthermore, an increasing number of centers worldwide use computed tomography (CT) scan which is less accurate than MRI assessment of onset during the acute phase of stroke. However, no specific method of dating cerebral ischemia is recommended to decide on thrombolysis in patients with stroke of unknown onset^5^. Thus, finding a new method complementary to MRI or CT scan could result in clear guidelines or recommendations for the evaluation of cerebral infarction of unknown onset. Dayon *et al*. observed the presence of high levels of two enzymes involved in oxidative stress, peroxiredoxin 1 (PRDX1) and glutathione s-transferase π (GST-π), in the blood of patients with cerebral infarction^6^. Moreover, GST-π levels were found to be associated with stroke onset in a prospective cohort of patients with acute ischemic stroke^3^. In the same cohort, we studied the kinetics of PRDX1 at the acute phase of cerebral infarction and its diagnostic performance to identify cerebral infarction of less than 3 and 6 hours. We also assessed the diagnostic performance of a PRDX1/GST-π panel using GST-π values determined during our previous study^3^.

## Material and Methods

### Patient inclusion

Patients from Geneva University Hospital were prospectively included over a period of two years (1^st^ December 2005 to 1^st^ January 2008).

The inclusion criteria were: age >18 years; diagnosis of cerebral infarction following neurological examination and cerebral imaging (MRI or CT scan); known and precise time of first symptoms within the first 24 hours following onset; and consent (from patient or relative) to take part in the study. The exclusion criteria were: pregnancy; known oncological disease; cirrhosis; renal failure; myocardial infarction or medical history of stroke of less than 3 months; ongoing treatment with neuroleptics and lithium; hemorrhagic stroke; subarachnoid hemorrhage; all cerebral traumatic lesions (including subdural, epidural, parenchymal hematoma and contusions). Inclusions depended on the presence of medical and paramedical staff in charge of the study (from Monday to Friday during usual working hours) and were consequently not consecutive.

A control group was constituted from patients with no current or history of cerebrovascular disease. They were healthy volunteers, patients admitted for limb fracture without neurological lesion, and patients from the Neurology Department admitted for multiple sclerosis, Parkinson’s disease or migraine.

### Data collection

Cerebral infarction was diagnosed following evaluation by a neurologist and time of stroke onset was precisely specified by the patient, relatives and/or first medical assistance interview. Demographic and clinical data including sex, age, vascular risk factors (hypertension, diabetes mellitus, tobacco consumption), medical history of atrial fibrillation and coronary heart disease, and National Institute of Health Stroke Score (NIHSS) at admission were collected. The type of diagnostic cerebral imaging (MRI or CT scan) performed was noted as well as the infarction location and volume assessed by the thresholding method. From these clinical and radiological data, cerebral infarctions were classified as total anterior circulation infarcts (TACI, cortical and subcortical involvement), partial anterior circulation infarcts (PACI, more restricted and predominantly with cortical involvement), lacunar circulation infarcts (LACI), and posterior circulation infarcts (POCI), following the criteria of the Oxfordshire Community Stroke Project Classification^7^. Use of reperfusion therapies was also noted.

Following inclusion, patients underwent blood withdrawals at different times: during the first 3 hours, between 3 and 6 hours, and after 6 hours following stroke onset. The number of blood withdrawals depended on the time of inclusion after stroke onset and the presence of the paramedical staff in charge of the study. Patients from the control group underwent only one blood withdrawal.

PRDX1 plasma levels were determined for each blood sample in the Department of Human Protein Sciences of the Medical Center of Geneva. GST-π plasma levels determined from the same blood samples of a previous study were used to assess the combined diagnostic performance of the PRDX1/GST-π panel^3^.

### PRDX1 quantification by ELISA

We used the PRDX1 ELISA kit (Abnova, Cat. No. KA0536) for the quantitative determination of human PRDX1 in the plasma samples. This assay is based on a sandwich ELISA. The 96-well plates provided were pre-coated with a monoclonal antibody specific to human PRDX1. Briefly, 100 μL of standards for calibration curves and homemade standards (in duplicates), and plasma samples (single analysis) diluted 1:5 were added to each well and the plates were incubated for 1 hour at room temperature followed by three washes with 300 μL of 1X wash buffer. Next, 100 uL of 1X “working AV-HRP solution” was added to each well and the plates were incubated for 30 minutes at room temperature followed by three washes with 300 μL of 1X wash buffer. Finally, 100 μL of substrate were added to each well turning the liquid blue. The plates were incubated for 10 minutes at room temperature. To stop the reaction, 100 μL of solution stop were added to each well turning the liquid yellow. The absorbance was measured at 450 nm within 20 minutes after the addition of the stop solution on the microplate reader FilterMax F3, Molecular Devices. All measurements were blinded. The reproducibility of the assay was assessed from standards by determining intra- and inter-run coefficients of variation.

### Statistical analysis

All analyses were carried out using IBM SPSS Statistics software, version 20 (SPSS Inc., Chicago, IL, USA). Continuous variables were described as median, mean ± standard deviation, categorical factors as frequency and percentage.

To identify differences of PRDX1 levels in the stroke patients *vs.* controls, the two groups were first compared for clinical characteristics and PRDX1 levels (overall, for each time window, and for each cerebral infarction subtype in the stroke patient group) using the Mann-Whitney U test, Chi-Square test and Fisher’s exact test when appropriate. PRDX1 levels were adjusted for clinical characteristics found to be statistically different. In the stroke patients, PRDX1 levels were also compared for those who had been treated with reperfusion therapies *vs.* those who had not using the Mann-Whitney U test. The following criteria were taken into account to identify an influence of the severity of the cerebral infarction on PRDX1 levels: NIHSS score at admission, infarction volume and cerebral infarction subtype. For the first two criteria, patients were divided into four quartiles and we used the Kruskal-Wallis test to detect significant differences of PRDX1 levels in one or several quartiles. The same analysis was used for cerebral infarction subtypes for the four groups (TACI, PACI, LACI and POCI).

To identify significant differences in PRDX1 levels in stroke patients (overall and in each cerebral infarction subtype) from blood samples withdrawn before *vs.* after 3 hours, and before *vs.* after 6 hours following onset, PRDX1 levels were dichotomized (<3 *vs.* >3 hours, and <6 *vs.* >6 hours) and compared using the Mann-Whitney U test.

To assess the diagnostic performance of PRDX1 to identify cerebral infarction of less than 3 hours and less than 6 hours, ROC analysis was performed to determine the area under the curve (AUC) and 95% interval confidence (95%CI), sensitivity (Se) and specificity (Sp) using the Youden index and related threshold. The same analysis was performed to assess diagnostic performance of the PRDX1/GST-π panel.

Significant difference was defined as p < 0.05.

### Ethics

This study was approved by the Ethics Committee of the N.A.C. (Neuclid, Apsic, Chirurgie) Department of the Geneva University Hospitals (study CER05–026 (05–058)). It was carried out in accordance with the principles of the Declaration of Helsinki. Each patient, or a legally authorized representative, was informed about the study and gave consent to participate.

## Results

Thirty-seven patients with ischemic stroke were included from whom 82 blood samples were withdrawn: 32 samples during the first 3 hours, 7 between 3 and 6 hours, and 43 after 6 hours following stroke onset. Cerebral infarction was diagnosed by MRI in 28 cases and CT scan in nine. One hundred and nine patients constituted the control group (4 healthy volunteers, 51 with various neurological diseases other than stroke, and 54 patients admitted for orthopedic conditions). The characteristics of stroke patients and the control population are summarized in Table 1. The only significant difference between the two groups was the prevalence of atrial fibrillation (p < 0.01).

Intra- and inter-run coefficients of variation of PRDX1 plasma level measurements were below 3% and 15% respectively.

**Figure 1:**
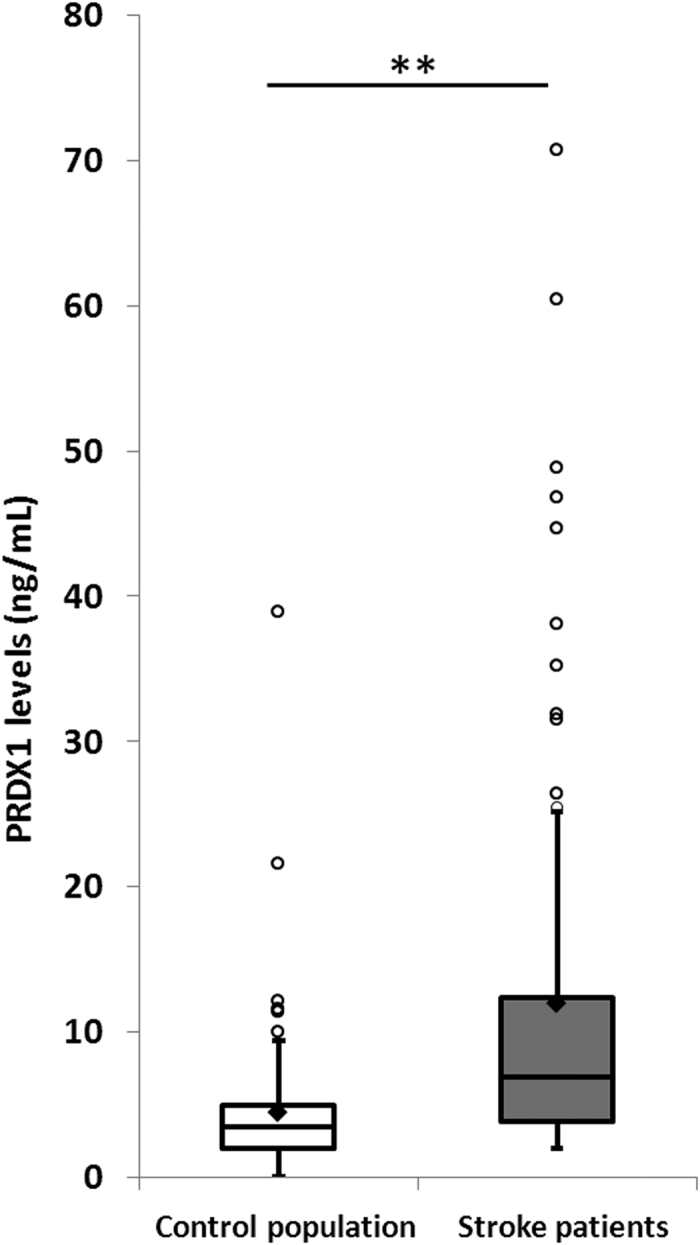
Peroxiredoxin 1 levels in the control population and stroke patients. Tukey’s box-and-whisker plots, box limits: interquartile range (IQR), middle line: median, diamond: mean, vertical lines: adjacent values (1st quartile −1.5 IQR; 3rd quartile +1.5 IQR), dots: outliers, peroxiredoxin 1 levels from 109 collections in control population and 82 collections from stroke patients. PRDX1: peroxiredoxin 1, **p < 0.01 Mann–Whitney U test.

**Figure 2:**
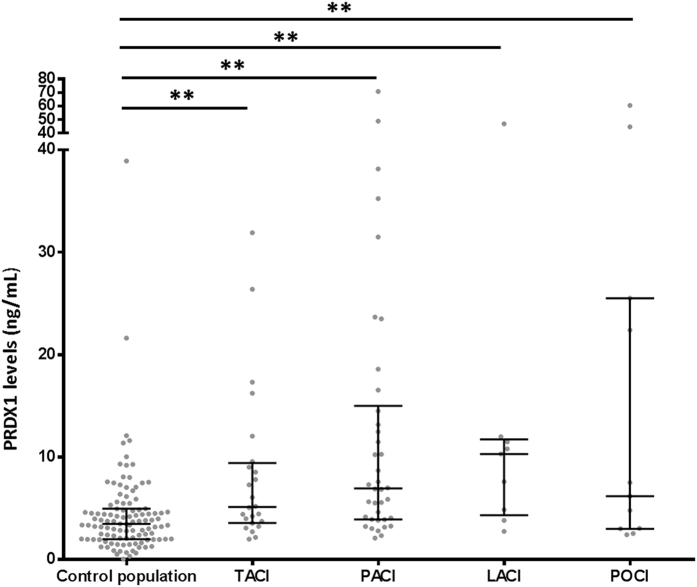
Peroxiredoxin 1 levels in the control population and in each cerebral infarction subtype. Grey dots: peroxiredoxin 1 plasma levels from each collection, middle line: median, upper and lower lines: interquartile range, TACI: total anterior circulation infarcts, PACI: partial anterior circulation infarcts, LACI: lacunar circulation infarcts, POCI: posterior circulation infarcts (Oxfordshire Community Stroke Project Classification), peroxiredoxin 1 levels from 109 collections in control population and 82 collections from stroke patients (24 samples in TACI, 38 samples in PACI, 9 samples in LACI, and 11 samples in POCI). PRDX1: peroxiredoxin 1, **p < 0.01 Mann–Whitney U test.

**Figure 3:**
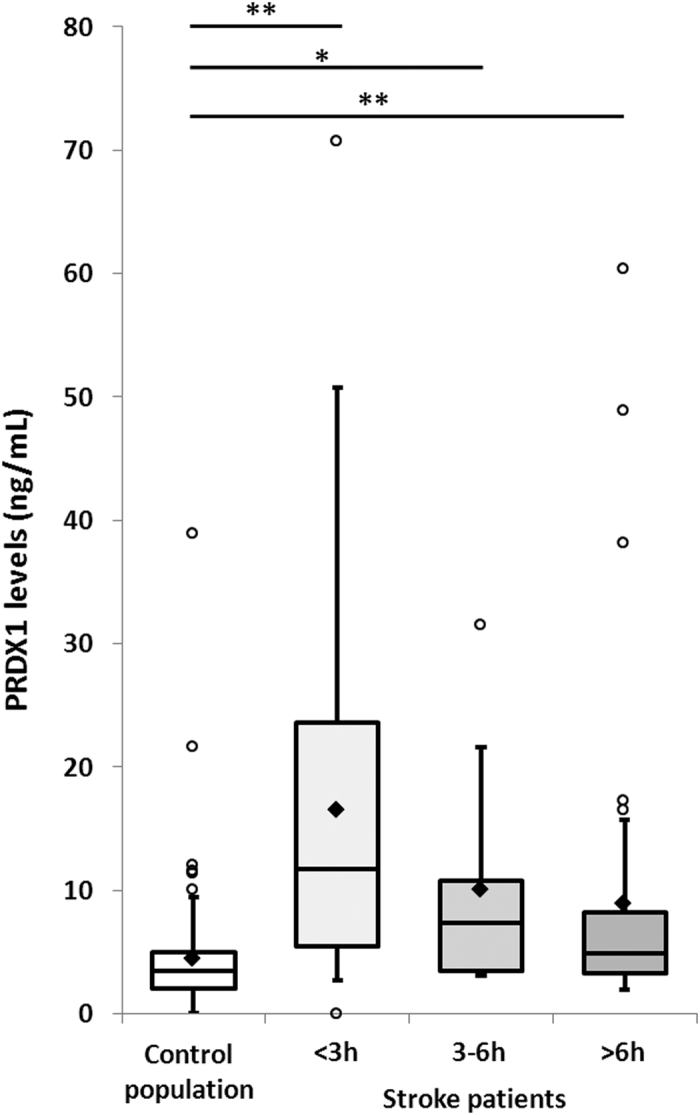
Peroxiredoxin 1 levels in the control population and stroke patients per time window. Tukey’s box-and-whisker plots, box limits: interquartile range (IQR), middle line: median, diamond: mean, vertical lines: adjacent values (1st quartile −1.5 IQR; 3rd quartile +1.5 IQR), dots: outliers, peroxiredoxin 1 levels from 109 collections in control population and 82 collections from stroke patients (32 samples during the first 3 hours, 7 samples between 3 and 6 hours, and 43 samples after 6 hours following stroke onset). PRDX1: peroxiredoxin 1, <3 h: within the first 3 hours, 3–6 h: between 3 and 6 hours, >6 h: after 6 hours following stroke onset, *p < 0.05, **p < 0.01 Mann–Whitney U test.

Overall PRDX1 median levels were significantly higher in stroke patients than in the control population: 6.9 ± 13.7 *vs.* 3.5 ± 4.5 ng/mL (p < 0.01) (Fig. 1, Table 2). This difference remained significant after adjustment for atrial fibrillation and for all cerebral infarction subtypes compared to the control population (Fig. 2). Median PRDX1 levels were significantly higher in the <3 hours (11.7 ± 15.6 ng/mL, p < 0.01), 3–6 hours (7.3 ± 10 ng/mL, p < 0.05), and >6 hours (4.9 ± 11.9 ng/mL, p < 0.01) time windows in stroke patients than in the control population (Fig. 3). PRDX1 levels were not different in the stroke patients treated with reperfusion therapies *vs.* not, for all samples (32 ± 5.1 *vs.* 50 ± 7.6 ng/mL, p = 0.19) and in each time window (data not shown). No significant difference in PRDX1 levels was found between the quartiles of stroke patients classified according to the NIHSS score at admission (p = 0.38), infarction volume (p = 0.13), or between the four groups of patients classified according to cerebral infarction subtype (p = 0.67).

**Figure 4:**
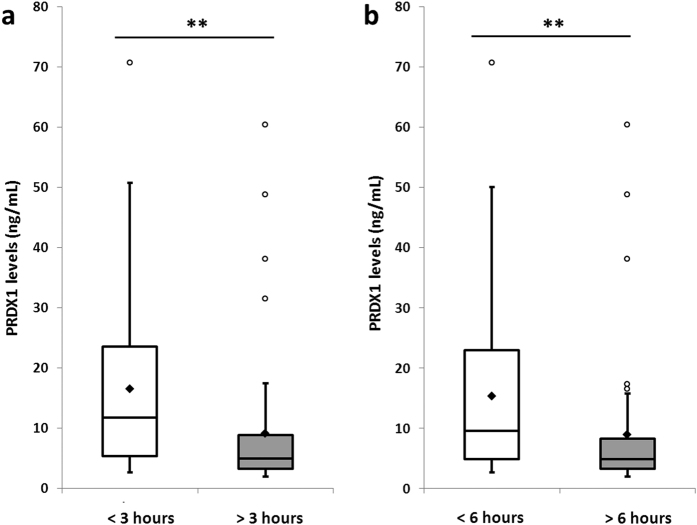
Peroxiredoxin 1 levels in stroke patients before and after 3 hours (**a**) and before and after 6 hours (**b**) following onset of cerebral infarction. Tukey’s box-and-whisker plots, box limits: interquartile range (IQR), middle line: median, diamond: mean, vertical lines: adjacent values (1st quartile −1.5 IQR; 3rd quartile +1.5 IQR), dots: outliers, peroxiredoxin 1 levels from 82 samples from stroke patients (32 samples before 3 hours, 50 samples after 3 hours, 39 samples before 6 hours and 43 samples after 6 hours). PRDX1: peroxiredoxin 1, <3 hours and >3 hours: before and after 3 hours following onset of cerebral infarction, <6 hours and >6 hours: before and after 6 hours after stroke onset, **p < 0.01 Mann–Whitney U test.

In the stroke patients, median PRDX1 levels were significantly higher in blood samples withdrawn before *vs.* after 3 hours following onset (11.7 ± 15.6 *vs.* 5 ± 11.6 ng/mL, p < 0.01) (Fig. 4a, Table 2) and in blood samples withdrawn before *vs.* after 6 hours following onset (9.6 ± 14.9 *vs.* 4.9 ± 11.9 ng/mL, p < 0.01) (Fig. 4b, Table 2). They also tended to be higher in each of the cerebral infarction subtype groups before *vs.* after 3 hours and before *vs.* after 6 hours but without reaching significance (Table 2).

**Figure 5:**
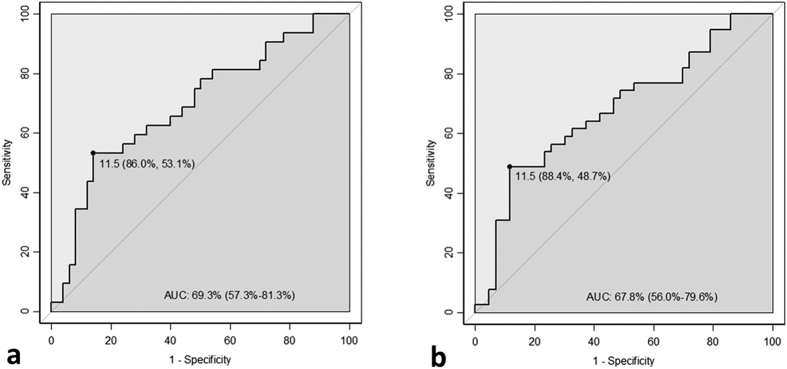
ROC analysis for diagnostic performance of peroxiredoxin 1 to identify cerebral infarction of less than 3 hours (**a**) and less than 6 hours (**b**) following onset of cerebral infarction. Results described as cut-off (ng/mL) determined with Youden index criterion, respective specificity and sensitivity, area under the curve (AUC) and 95% confidence interval.

The diagnostic performance of PRDX1 levels to identify cerebral infarction of less than 3 and 6 hours are represented by the ROC curves in Fig. 5. ROC analysis gave an AUC of 69% (95%CI: 57–81%), an Se of 53% and an Sp of 86% for identifying a cerebral infarction of less than 3 hours and an AUC of 68% (95%CI: 56–80%), an Se of 49% and an Sp of 88% for a cerebral infarction of less than 6 hours (Fig. 5a,b, Table 2). ROC analysis of the PRDX1/GST-π panel failed to show that the addition of GST-π levels improved the diagnostic performance of PRDX1 for identifying cerebral infarction of less than 3 hours (AUC = 66% (95%CI: 49–82%), Se = 56% and Sp = 75%) or less than 6 hours (AUC = 67% (95%CI: 54–81%), Se = 67% and Sp = 67%).

## Discussion

The results of our study show that PRDX1 levels are high during the first hours following stroke onset suggesting that it could play a role in identifying patients with cerebral infarction who fall within the therapeutic window for reperfusion therapies. One third of patients with cerebral infarction are admitted to hospital with unknown time of onset^8^,^9^. Thus, they cannot benefit from thrombolysis which is considered as one of the main treatments to improve outcome at the acute phase of ischemic stroke. Reperfusion therapies administered after the accepted cut-off expose patients to serious hemorrhagic transformation. The main objective of a method to accurately specify the time of the stroke is therefore to exclude with certainty patients who do not fall within this therapeutic window. This means the highest Sp is required for a method to identify patients within the therapeutic window. The current radiological method of estimating stroke onset time (diffusion/FLAIR mismatch) has been evaluated by Thomalla *et al*.^4^ who found an Se of 62% and an Sp of 78% to identify cerebral infarction of less than 4.5 hours and an Se of 56% and an Sp of 87% for less than 6 hours. Diagnostic performances of PRDX1 are comparable even if we worked on a window of less than 3 hours instead of 4.5 hours. The identification of proteins with kinetics associated with cerebral infarction onset could improve the diagnostic performance of cerebral MRI by means of a combined strategy. Turck *et al*. have previously described the ability of another enzyme to determine stroke onset, GST-π, also involved in oxidative stress. They found a slightly higher performance than PRDX1 to identify a cerebral infarction of less than 3 hours (Se = 68% and Sp = 82%)^3^. However, our study contributes important data about the 6-hour window – a time limit usually applied to make decisions about endovascular treatment with mechanical thrombectomy – which was not described in the GST-π study. We demonstrate that PRDX1 maintains its timing ability at 6 hours. We failed to demonstrate an improved diagnostic performance by combining PRDX1 with GST-π. This was most probably because of the sample size of our study, but we can also hypothesize that the functions of these two biomarkers during stroke are too similar and concurrent to be complementary in estimating time of cerebral infarction.

**Figure 6:**
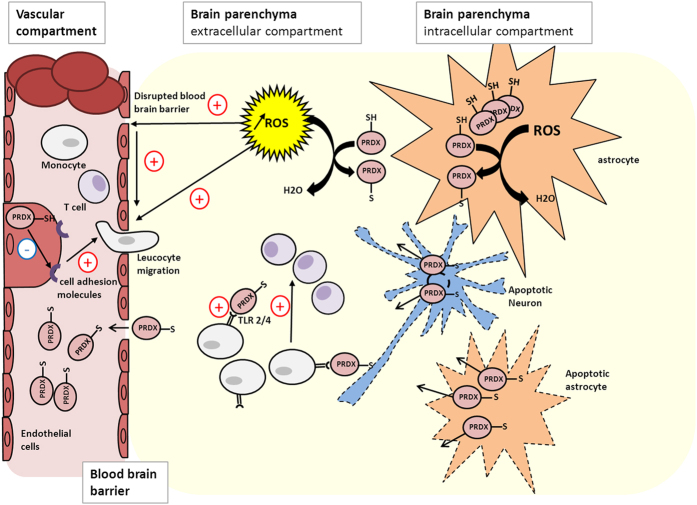
Different roles of peroxiredoxins during cerebral infarction. During cerebral ischemia, peroxiredoxins reduce reactive oxygen species into less reactive components and inhibit expression of cell adhesion molecules in endothelial cells promoting leukocyte migration in the brain parenchyma. However, the oxidized form produced and released from apoptotic cells can activate monocytes and promote inflammation in the brain parenchyma. High serum levels of peroxiredoxins would result from production in endothelial cells and diffusion from the extracellular into the vascular compartment through disrupted blood brain barrier. PRDX: peroxiredoxin, TLR 2/4: toll like receptors 2 and 4, ROS: reactive oxygen species.

PRDX-1 and GST-π belong to the antioxidant enzymes involved in the neutralization of reactive oxygen species (ROS) resulting from oxidative stress^10^,^11^,^12^. Their induction and activation at different stages of cerebral infarction and inflammation, mainly described for peroxiredoxins, explain the high serum levels observed in stroke patients by Dayon *et al*. and in our study^6^. ROS are responsible for alteration of membrane lipids, proteins and DNA^13^. Their production rises with cerebral ischemia and even during reperfusion promoting neuronal death^14^. In this context, antioxidant enzymes convert ROS into less reactive components^15^. Peroxiredoxins are abundant and ubiquitous proteins with a cysteine residue near the N-terminal portion which interact with ROS and reduce their activity. The gene encoding PRDX1 is located on the human chromosome 1p34^16^. Ischemia would induce PRDX1 production in the brain in response to oxidative stress to reduce ROS and enhance production of antioxidant agents^17^. It has been suggested that induction of peroxiredoxins reduces the ischemic area in animal models of stroke^18^. Moreover, in models of cerebral inflammation, PRDX1 induced by oxidative stress protects the integrity of the blood brain barrier and prevents expression of cell adhesion molecules which mediate leucocyte migration into the parenchyma^19^. On the contrary, oxidized peroxiredoxins released into extracellular spaces following neuronal death enhance inflammation inherent to stroke and are held responsible for extension of infarction^20^. They act as damage-associated molecular pattern molecules activating macrophage through toll like receptors 2 and 4, then recruit T cells and finally maintain the inflammation cascade (Fig. 6)^21^. Overall, whether their actions are favourable or not, these data support increased levels of PRDX1 during cerebral ischemia. The presence of PRDX1 in the blood would be the result of a direct production by endothelial cells, diffusion from the cerebrospinal fluid and the extracellular space due to disruption of blood brain barrier^3^,^6^. PRDX1 production is usually described as a secondary reaction to cerebral infarction occurring 12 hours after onset^21^. However, in our study, the highest levels of PRDX1 were found in the first 3 hours followed by a decrease.

PRDX1 plasma levels in the stroke patients overall were significantly higher in samples withdrawn before 3 hours and before 6 hours than those withdrawn after these time intervals. They tended to be higher for all the cerebral infarction subtypes (TACI, PACI, LACI and POCI), but this absence of significance is almost certainly due to lack of power. It is worthwhile emphasizing that patients with LACI, characterized by low volume, present PRDX1 levels comparable to other cerebral infarction subtypes and significantly higher than controls. These patients are prone to present leukoaraiosis corresponding to chronic ischemia of brain tissues which may increase related biomarker levels. Particular attention should be paid to patients with LACI in further studies as biomarker levels may not specifically represent recent infarction. Although we did not find a significant increase in biomarker levels in patients with elevated stroke volume and NIHSS score, we believe that the severity of a stroke assessed by clinical and radiological criteria has to be taken account in interpreting biomarker levels. Logically, infarction volume would be more likely to influence biomarker levels. The NIHSS score, as clinical severity criteria, is mainly related to infarction volume in TACI and PACI, but less so in LACI and POCI where small infarctions can lead to severe neurological deficiency.

We recognize some limitations of this first study. The studied time windows do not allow us to conclude on the diagnostic performance of PRDX1 to identify cerebral infarction of less than 4.5 hours which is the therapeutic window of intravenous thrombolysis for most patients suffering from ischemic stroke. Furthermore, the sample size was small and we had insufficient human resources to systematically withdraw blood for each time window and in particular between 3 and 6 hours where we had few samples. This mainly explains the wide distribution of PRDX1 values in some subgroups and the lack of power to reach significance for cerebral infarction subtypes when comparing biomarker levels from one time window to another. Moreover, it prevented us from demonstrating that combining PRDX1 and GST-π levels results in better assessment of time of stroke onset even though a biomarker panel would be the best strategy to improve diagnostic performance. In the same way, we failed to show the impact of stroke severity on PRDX1 levels as our study included very few patients with very high stroke volume (only two patients >60 mL) with fairly similar median volumes between TACI and PACI. Important radiological data, such as diffusion/FLAIR mismatch, were not collected and some patients were examined with cerebral CT scan leading to inaccurate assessment of stroke volume. We also recognize that the heterogeneity of the control population represents a limitation. Nevertheless, this population consisted of subjects typically examined in neurovascular practice and classified as “stroke mimics”. These represent conditions other than those of neurovascular origin with clinical presentation similar to stroke (migraine, multiple sclerosis, osteoarticular and psychiatric causes…). For the purpose of diagnosis it was therefore of interest to assess if PRDX1 levels were significantly different in these patients compared to those with cerebral infarction.

To address these issues, we are planning to conduct further studies with more patients, standardized cerebral imaging (consisting of cerebral MRI with assessment of infarction volume, diffusion/FLAIR mismatch and penumbra), and systematic blood withdrawals in different and narrower time windows. We will also assess the influence of severity criteria on biomarker levels. Furthermore, we would differentiate between healthy volunteers and patients with stroke mimics in the control population. As mentioned previously, the main objective of biomarkers to determine stroke time-of-onset would be to exclude with certainty patients outside therapeutic window due to the risk of hemorrhagic transformation. For this purpose, studies with a higher number of patients will enable us to assess diagnostic performance of biomarkers in ROC analysis at the highest level of Sp of 100%, in other words to assess the ability of biomarkers to diagnose cerebral infarctions within therapeutic window after total exclusion of those are not. Furthermore, it would be of high interest to combine the diagnostic performance of PRDX1 and GST-π along with other proteins released or activated at different times during cerebral ischemia and inflammation. In this way, the diagnostic performance of necrosis markers such as S100b^22^, peptides from hormonal stress response such as copeptin^23^, or other proteins involved at different stages of inflammation such as cell adhesion molecules will be explored^24^. In addition, a combination of a future biologic method with the radiological diffusion/FLAIR mismatch method could represent the most interesting panel. Nevertheless, forthcoming therapeutic strategies will not only be based on infarction time-of-onset but also on the presence of viable tissue with oligemia^25^. This depends on collateral circulation and is usually assessed by cerebral MRI with diffusion/perfusion-weighted imaging mismatch^26^. Accordingly, we will have to identify biomarkers that certify and quantify penumbra. Finally, the practical application of a biologic time estimation method to safely administer reperfusion therapies to patients with ischemic stroke of unknown onset will not be possible without rapid bedside evaluation of biomarkers levels. To meet this requirement, we plan to introduce a rapid decentralized method of protein measurement^27^.

In conclusion, this analysis of the kinetics of PRDX1, involved in oxidative stress during stroke, show that it could form the basis of a biologic method of estimating time of cerebral infarction. These preliminary results have to be confirmed in a larger study. Moreover, other biomarkers of cerebral infarction and inflammation should be explored to complete the findings. A combination of biologic and radiologic approaches could constitute a way of identifying stroke patients who can safely be administered reperfusion therapies. This would lead to recommendations for the evaluation of patients with ischemic stroke of unknown onset.

## Additional Information

**How to cite this article**: Richard, S. *et al*. Diagnostic performance of peroxiredoxin 1 to determine time-of-onset of acute cerebral infarction. *Sci. Rep.* **6**, 38300; doi: 10.1038/srep38300 (2016).

**Publisher's note:** Springer Nature remains neutral with regard to jurisdictional claims in published maps and institutional affiliations.

## Figures and Tables

**Table 1:** Population characteristics.

Characteristics	Control population (n = 109)	Stroke patients (n = 37)	p value
**Age (years)**			
Median (range)	69 (26–88)	71 (43–89)	0.13^a^
**Female sex**			
n (%)	48 (44%)	16 (43%)	0.93^b^
**NIHSS score**			
Median (range)	—	8 (0–21)	—
**Atrial fibrillation**			
n (%)	6 (5%)	16 (43%)	<0.01^b^
**Diabetes mellitus**			
n (%)	14 (12%)	4 (11%)	0.99^c^
**Dyslipidemia**			
n (%)	20 (18%)	6 (16%)	0.77^b^
**Hypertension**			
n (%)	51 (47%)	21 (56%)	0.29^b^
**Myocardial infarction**			
n (%)	2 (2%)	1 (3%)	0.99^c^
**Tobacco**			
n (%)	42 (38%)	18 (49%)	0.28^b^
**Infarction subtype**			
TACI n (%) – median volume	—	12 (32%) – 35 mL	—
PACI n (%) – median volume	—	17 (46%) – 10 mL	—
LACI n (%) – median volume	—	4 (11%) – 1 mL	—
POCI n (%) – median volume	—	4 (11%) – 7 mL	—
**Reperfusion therapies**			
n (%)	—	15 (40%)	—

NIHSS: National Institute of Health Stroke Scale, TACI: total anterior circulation infarcts, PACI: partial anterior circulation infarcts, LACI: lacunar circulation infarcts, POCI: posterior circulation infarcts (Oxfordshire Community Stroke Project Classification), a: Mann–Whitney U test, b: Chi—Square test, c: Fischer’s exact test.

**Table 2:** Peroxiredoxin 1 performances to diagnose and to time cerebral infarction.

**Control population** ***vs.*** **Stroke patients**	**Stroke patients** median level ± standard deviation n = 82	**6.9** ± **13.7 ng/mL**	**p** < **0.01**
	**Control population** median level ± standard deviation n = 109	**3.5** ± **4.5 ng/mL**	
**Diagnostic performance: identification of cerebral infarction <3 hours**	**Samples <3 hours** median level ± standard deviation Overall n = 32	**11.7** ± **15.6 ng/mL**	**p** < **0.01**
	TACI n = 11	8.6 ± 9.7 ng/mL	p = 0.1
	PACI n = 14	10.4 ± 18.2 ng/mL	p = 0.14
	LACI n = 4	11.7 ± 19,5 ng/mL	p = 0.28
	POCI n = 3	25.5 ± 12.1 ng/mL	p = 0.08
	**Samples >3 hours** median level ± standard deviation Overall n = 50	**5** ± **11.6 ng/mL**	
	TACI n = 13	4.4 ± 4,1 ng/mL	
	PACI n = 24	6.4 ± 12 ng/mL	
	LACI n = 5	7.6 ± 3 ng/mL	
	POCI n = 8	4 ± 19.9 ng/mL	
	**ROC analysis** AUC (95%CI), Sensitivity, Specificity	69% (57–81%), 53%, 86%	
**Diagnostic performance: identification of cerebral infarction <6 hours**	**Samples <6 hours** median level ± standard deviation Overall n = 39	**9.6** ± **14.9 ng/mL**	**p** < **0.01**
	TACI n = 13	8.5 ± 9.2 ng/mL	p = 0.15
	PACI n = 18	10 ± 16.8 ng/mL	p = 0.07
	LACI n = 4	11.7 ± 19.5 ng/mL	p = 0.28
	POCI n = 4	24 ± 17 ng/mL	p = 0.07
	**Samples >6 hours** median level ± standard deviation Overall n = 43	**4.9** ± **11.9 ng/mL**	
	TACI n = 11	4.4 ± 4.3 ng/mL	
	PACI n = 20	5.7 ± 12.2 ng/mL	
	LACI n = 5	7.6 ± 3.1 ng/mL	
	POCI n = 7	4.8 ± 21.2 ng/mL	
	**ROC analysis** AUC (95%CI), Sensitivity, Specificity	68% (56–80%), 49%, 88%	

PRDX1: peroxiredoxin 1, TACI: total anterior circulation infarcts, PACI: partial anterior circulation infarcts, LACI: lacunar circulation infarcts, POCI: posterior circulation infarcts (Oxfordshire Community Stroke Project Classification), 95% CI: 95% confidence interval, n: number of plasma samples, p: Mann–Whitney U test p value comparing PRDX1 levels in control population *vs.* stroke patients, in plasma samples <*vs.* > 3 hours, and <*vs.* > 6 hours after stroke onset.
